# Addressing the Problem of Lysine Glycation Prediction in Proteins via Recurrent Neural Networks

**DOI:** 10.1155/bmri/2426944

**Published:** 2025-09-15

**Authors:** Ulices Que-Salinas, Dulce Martinez-Peon, Gerardo Maximiliano Mendez, P. Argüelles-Lucho, Angel D. Reyes-Figueroa, Christian Quintus Scheckhuber

**Affiliations:** ^1^ Earth Sciences Center, Veracruz University, Xalapa, Mexico, uv.mx; ^2^ Department of Electrical and Electronic Engineering, National Technological Institute of Mexico, Guadalupe, Mexico; ^3^ Department of Systems and Computing, National Technological Institute of Mexico, Veracruz, Mexico; ^4^ CONACYT, Mexico City, Mexico, conacyt.mx; ^5^ Applied Mathematics Department, Mathematics Research Center, Apodaca, Mexico; ^6^ School of Engineering and Sciences, Tecnologico de Monterrey, Monterrey, Mexico, tec.mx

**Keywords:** amino acid properties, classification, glycation, lysine, recurrent neural network

## Abstract

A distinguishing feature of the metabolic disorder diabetes involves elevated damage to cellular components. Glycation, in contrast to glycosylation, is regarded as a strictly nonenzymatic process that involves the reaction of sugars (e.g., glucose and fructose) and sugar‐derived molecules (e.g., methylglyoxal) with amino groups of biologically highly relevant molecules, such as nucleic acids, lipids, and proteins. The primary form of alteration arises from the chemical interaction between glycating agents and proteinaceous arginine/cysteine/lysine residues. Glycation may result in the formation of advanced glycation end‐products (AGEs) which are mostly detrimental and compromise the function of the target molecule irreversibly. There are no clear sequence motifs in proteins that allow a straightforward identification of potential glycation sites. However, the physicochemical properties of amino acids that flank the glycated residue seem to play a key role in determining if glycation occurs or not. Here, we used a curated version of the CPLM database to implement a recurrent neural network strategy for the classification of lysine glycation to better understand which of eight physicochemical properties might influence glycation more than others. By using the most promising property for the characterization of amino acids next to lysine sites, isoelectric point, it was possible to obtain a 59.6% accuracy for correctly predicting lysine glycation. When the properties mass and torsion angle were used together, the accuracy increased to approximately 60%. Overall, our approach contributes to the understanding of glycation principles and can aid the task of narrowing down possible sites of lysine glycation in protein targets for further analysis.

## 1. Introduction

Proteins are constructed from a rather small set of unique amino acids. The roster of amino acids comprises 20 “standard” ones, along with a few more esoteric proteinogenic variants, such as selenocysteine and pyrrolysine [1]. This allows for an astronomical range of individual potential sequences, even in relatively short proteins. In addition to this staggering diversity, the plethora of posttranslational modifications (PTM) that amino acids can undergo should be considered. These modifications introduce layers of regulation and control, encompassing an array of processes leading to adducts. Notable examples include acetylation [2], phosphorylation [3], methylation [4], and ubiquitination [5, 6]. The PTM of specific amino acids can occur enzymatically or through nonenzymatic means. For instance, glycosylation, vital for protein sorting, secretion, and cellular recognition is accomplished by N‐ and O‐glycosyltransferases and other enzymes [7]. Another critical example involves the reversible modification of histones by histone acetylases and deacetylases, a fundamental process for the coordinated regulation of gene expression [8].

In contrast, glycation is a predominantly nonenzymatic process, entailing the reaction of sugars (e.g., glucose and fructose) and sugar‐derived compounds with biologically significant molecules, such as nucleic acids, lipids, and proteins [9]. First, a Schiff base between the target and the glycating agent is formed, which is rearranged to yield an Amadori product. Typically, these reactions culminate in the formation of advanced glycation end‐products (AGEs), most of which exert detrimental effects and irreversibly compromise the function of the target molecules [10, 11].

Within proteins, the side chains of lysine and arginine emerge as primary targets for AGE formation [12, 13]. Methylglyoxal (MGO), a highly reactive glycating compound, ranks among the most prolific culprits. It arises as a toxic by‐product during metabolic processes, such as glycolysis, in which it is produced by spontaneous elimination of phosphate from the glycolytic intermediate dihydroxyacetone phosphate [14]. Under physiologically normal circumstances, cellular MGO levels remain relatively low, typically hovering around 0.3–6 *μ*M [15]. This is maintained by dedicated enzymatic defense systems, such as glyoxalase I and II, aldose reductases, and low‐molecular‐weight scavengers [16, 17]. However, in certain pathological conditions (e.g., atherosclerosis, diabetes, neurodegeneration, and cancer) and in aging cells and tissues, MGO can pose challenges to cellular viability due to heightened production or hindered removal [18–20]. It is important to note that MGO‐mediated protein modifications can play a pivotal role in various signaling processes and gene regulation. This has been substantiated through dedicated studies, often conducted in simple eukaryotic model systems known for their experimental tractability [21]. On the other hand, biochemical characterization of human hemoglobin revealed that several factors determine the outcome of glycation, such as the absence of aspartate or glutamate residues, which could inhibit glycation by forming electrostatic interactions, the presence of histidine residues to facilitate acid‐base catalysis in the Amadori rearrangement, and an amino acid residue that can stabilize a phosphate during proton transfer [22]. A recent study sheds more light on the mechanisms governing peptide glycation suggesting that there is no general correlation between the amino acid content and the susceptibility towards the initial phase of glycation reactions [23]. The authors state that a pronounced contribution of other factors such as the amino acid sequence, i.e., the amino acid microenvironment, determines whether glycation of a peptide might occur. However, despite extensive research on the importance of MGO binding to specific amino acids in target proteins, it has become evident that there is no straightforward consensus sequence for reliably predicting potential glycation sites [24]. Still, several promising approaches have been made that allow for the prediction of potential lysine glycation sites. Examples include GlyNN [25], which employs an artificial neural network (ANN) [26] to predict lysine glycation, and BPB_GlySite [27], PreGly [28], PredGly [29], Gly–PseAAC [30], Glypre [31], iProtGly–SS [32], GlyStruct [33], BERT‐Kgly [34], and iGly‐IDN [35]. These methods employ approaches such as bi‐profile Bayes features extraction, position‐specific amino acid propensity, transformers (BERT)–based models and models trained with support vector machine (SVM) classifiers. Traditionally, lysine modification has been the focus of extensive research, leading to the availability of a comprehensive database of lysine modifications, such as PLMD [36], based on CPLM [37] and CPLA 1.0 [38]. For the detection of arginine‐dependent glycation, a predictor based on ANNs that utilizes short peptides that were subjected to controlled in vitro glycation, yielding a small but high‐quality data set, was recently published [24, 39]. The newest version of the CPLM database (4.0) is built on rigorous manual curation and high‐throughput experimental evidence, focusing on experimentally verified lysine PTMs [40]. It has been successfully utilized to study PTMs in different contexts such as proteomics for global mapping of functional lysines on the cell surface of HeLa cells [41], deciphering the functional roles of protein succinylation and glutarylation [42], and small‐sample learning to reveal propionylation in determining global protein homeostasis [43] to give three examples from recent research. The creators of CPLM 4.0 collected data through extensive literature mining, primarily from PubMed, and only included lysine modifications supported by experimental validation—mainly from mass spectrometry (MS)–based proteomics studies. To enhance detection sensitivity, many of these studies employed techniques such as immunoprecipitation using PTM‐specific antibodies, stable isotope labeling (e.g., SILAC), and various enrichment methods like affinity chromatography or chemical tagging. CPLM 4.0 integrates data from a broad range of biological sources, covering key model organisms such as humans (*Homo sapiens*), mice (*Mus musculus*), yeast (*Saccharomyces cerevisiae*), and the plant *Arabidopsis thaliana*. The database reflects findings across diverse cell types, including immortalized human cancer cell lines (e.g., HeLa and HEK293), stem cells, primary tissues such as liver and brain, and microbial cultures, offering wide biological and experimental coverage for the study of lysine PTMs. The type of glycation reported in CPLM 4.0 is predominantly MGO‐derived, as this is the reagent used in most of the published studies due to its reactivity compared to glucose, which facilitates detection. This does not exclude that other types of glycation (e.g., glucose‐derived) are to a lesser extent present in the database as well.

In conclusion, it is relevant to narrow down potential sites of protein modification, as the experimental demonstration of specific amino acid modifications can be prohibitively expensive in terms of time, resources, and labor, particularly in larger proteins housing numerous potential glycation sites. Our aim was to test whether it is possible to predict lysine glycation sites in proteins by using eight physicochemical properties of neighboring amino acids in conjunction with a recurrent neural network (RNN). This approach lets us identify properties that are more relevant than others for successful glycation. We use a high‐quality protein lysine modification data set to employ the RNN to classify lysine glycation. It shows that when using the isoelectric point (IEP), a 59.6% accuracy for lysine glycation is obtained. Additionally, when combining the two properties, mass and torsion angle (ToA), the accuracy increases to 59.9%.

## 2. Materials and Methods

### 2.1. General Outline of the Experimental Approach

**Figure 1:**
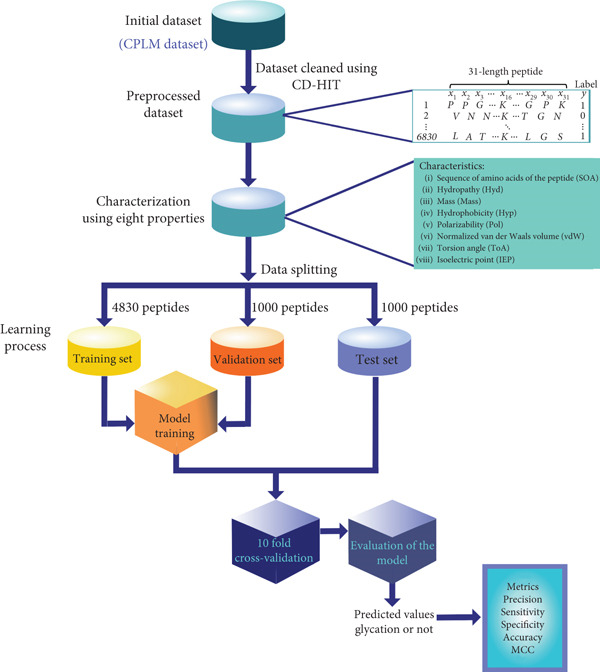
Flowchart of the steps to classify glycation of lysine. The data collected from the CPLM 4.0 database was processed using the CD‐HIT tool, forming arrays of vectors characterized by the physicochemical properties of the amino acids. The processed database was divided into three sets (training, validation and testing) to perform the RNN learning process and test the results obtained through a series of metrics.

Figure 1 shows the methodology that was followed. We utilized a previously published database (CPLM) [40] and its derivative developed by Liu et al. [34] which contains *H. sapiens* protein sequences. Redundant information from CPLM was removed by these authors utilizing CD‐HIT [44] with a 30% cut‐off. This cut‐off value has been successfully applied in previous projects, such as the functional classification of immune regulatory proteins [45] and the prediction of protein N‐Glycosylation [46]. The 10 folders of the lysine glycation data from Liu et al. are publicly available from https://github.com/yinboliu-git/Gly-ML-BERT-DL. Using the test and training data sets, we performed a 10‐fold cross‐validation. It should be noted that in these data sets, positive and negative glycation sites are clearly labeled and balanced. Lysine residues are classified as “nonglycated” based on the empirical absence of detectable modifications in experimental data. Specifically, if a lysine‐containing peptide is successfully identified by MS and mapped within a protein, but no mass shift corresponding to glycation is detected at that site, the lysine is considered nonmodified in that experimental context. However, this classification only applies to lysines located in regions of the protein that are confidently covered by peptide mapping. Lysines found in regions with low or no coverage are excluded from this designation to prevent false negatives, as their modification status cannot be reliably assessed. Therefore, the label “nonglycated” reflects the absence of observed modification under specific conditions, not a definitive lack of modifiability. Eventually, 6830 peptides in total, each corresponding to a sequence of 31 amino acids and containing a central lysine residue that was reported to be glycated or not, were obtained.

Historically, most of the papers in this field use only position‐specific amino acid propensity; here, we proposed a study based on the amino acid microenvironment, using eight physicochemical properties of the amino acid neighbors: the proper sequence of amino acids, that is, the structure of the amino acid sequence (SoA) of the peptide, hydropathy (Hyd), mass, hydrophobicity (Hyp), polarizability (Pol), normalized van der Waals volume (vdW), ToA, and IEP (Table S1). These amino acid sequences were used to feed a numerical algorithm based on RNNs, which was utilized as a model for the intelligent recognition process of the presence of glycation in lysine. As the final stage of the methodology, the trained model was validated on an independent test set. Therefore, the following metrics were determined as quantitative parameters to evaluate the performance of the model: (i) accuracy, the ratio between the correct predictions for both true glycated and true nonglycated proteins; (ii) precision, a ratio based on the prediction of true glycated proteins and the sum of true and false positive glycation predictions; (iii) sensitivity, a ratio for the prediction of true glycated proteins based on true positive and false negative glycation predictions; (iv) specificity, a ratio between the prediction of true nonglycated proteins based on the true and false predictions of nonglycation; (v) the Matthews correlation coefficient (MCC), a metric that measures the correlation between the model and the real system; and (vi) the ROC (receiver operating characteristic) curve [34, 47]. Although these metrics have values ranging from 0 to 1, representing a 1 that the model correctly predicts 100% of the real values, it should be considered that in the case of MCC, the values range from −1 to 1, which means perfect misclassification or classification, respectively. At the same time, MCC = 0 is the expected value for the “coin tossing” classifier. It is worth mentioning that MCC is the only metric that considers whether the classifier was able to correctly predict both the majority of positive and negative data instances [47]. To quantify the amino acid microenvironment, the symbolic letters that represent the amino acids of the 6830 peptides were replaced by a numerical value linked to a particular physicochemical property (see Table S1). For each peptide, eight different vectors of 31 numerical values were generated; we studied the neighborhood of 10 amino acids around the lysine (before and after), each one of them representing one of the physicochemical properties previously discussed.

### 2.2. Characterization Scheme

First, the symbolic letters that represent the amino acids of the 6830 peptides were replaced by a numerical value linked to a particular physicochemical property (see Table S1). For each peptide, eight different vectors of 31 numerical values were generated, each one of them representing one of the physicochemical properties previously discussed. Thus, the vectors maintain the same order of the amino acids but are now represented by the numerical value corresponding to one of these properties. Therefore, each of the 6830 peptides used for this study will be represented by eight vectors of 31 numerical values.

For our numerical model learning process, the samples were divided in the following way: 4830 sequences for training, 1000 peptides were used for the internal learning validation process, and the remaining 1000 were used to verify the performance of the model through an independent test. For the training step, a RNN using the characterized data with the eight properties SoA, Hyd, mass, Hyp, Pol, vdW, ToA, and IEP was obtained [39].

Importantly, after the cutoff process using CD‐HIT described above, the 6830 peptides are balanced with respect to positive or negative glycation sites. As such, it was not necessary to employ oversampling or undersampling techniques such as random oversampling. A table containing the number of glycation sites is shown in the Table S2.

### 2.3. RNN Architecture

**Figure 2:**
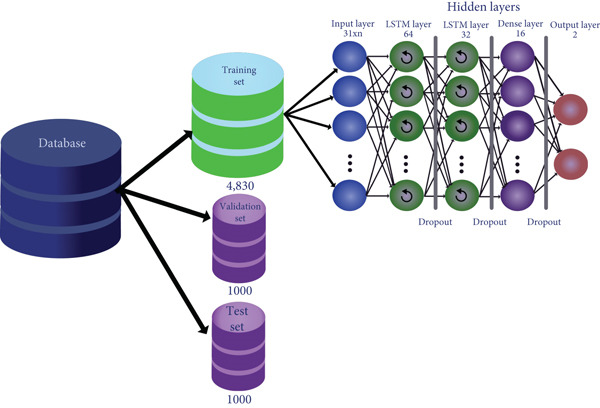
Structure of the RNN. Once the database has been divided into three sets, the training set is used for the learning process of the RNN. This consisted of two hidden LSTM‐type recurrence layers and a dense layer followed by the output layer of the results. This process is shown from left to right, indicating at the top of each RNN layer the number of neurons that compose it.

The structure of the RNN used was of the long short‐term memory (LSTM) type. It contains an input layer, a hidden layer, and an output layer, as shown in Figure 2. The output layer has two neurons, one to indicate the probability of the presence of glycation on the central amino acid (i.e., lysine) and the other for the opposite. The outcome of the RNN was determined by considering which of the two options presents a higher probability, repeating the process a total of 20 times to ensure the reproducibility of the model. The hidden layer is made up of six distinct layers. The first contains 64 neurons; each neuron is an LSTM unit with a tangent hyperbolic function and a linear regularized l2 type, followed by a dropout layer. After that, there is another layer of 32 LSTM units with the same configuration as those used in the first hidden layer. This is followed again by a second dropout layer that contributes to forgetting the adjusted weights to avoid overtraining. After that, a dense layer is highly connected, composed of 16 neurons with a rectified linear unit (ReLU) activation function followed by a dropout layer. Finally, the input layer consists of an arrangement of neurons that receives the internal values of an array of 31X2 elements for Cases 1 and 2 and 31X8 for Case 3 (see below for the description of the cases).

For the RNN, a sparse categorical cross‐entropy cost function with an ADAM type optimizer was used, and the accuracy metric to monitor the internal results was determined [48]. For the training, a batch size of 64 was applied, with several training epochs that depended on an early stopping algorithm: if the accuracy after 20 epochs does not increase compared with the validation set, the training stops, and the epoch that contains the better results is maintained. Due to the early stopping algorithm, the number of training epochs varied approximately between 30 and 120.

The parameters used by the RNN were tuned considering a 10‐fold cross‐validation. To achieve this, we divided the data into 10 groups of equal size and we implemented the algorithms for the first group of data to obtain the results. We repeated this nine more times with the other groups. The parameters that showed the best performance were the ones used for the independent test. Here, it is important to highlight that considering the random initiation of some processes (such as weights), it was considered pertinent to average over 20 training processes of the RNN.

Subsequently, the learning of the RNN was achieved with the training dataset using a set of validation to monitor the performance and to follow the end of the process, reviewing for this purpose the values of some metrics (such as accuracy) throughout the training evolution. Three different cases were defined to test the efficiency of the model and achieve the objective of this study:

**Case 1.**

For each of the eight properties, a matrix of length 31X2 was built, where 31 is the length of the peptide and contains the obtained values of each property for each amino acid of the peptide, the mass, for example. This was duplicated for the second column. The approach allows for the analysis of the effect of each of the physicochemical properties of the amino acids separately.

**Case 2.**

Combinations of two properties for the input of the RNN. The input size was 31X2, where the first column belongs to the values of one property and the second column belongs to the values of a second property; for the same peptide, the total number of combinations was 28. This second case allows for determining the weight of the combinations of physicochemical properties in the glycation process.

**Case 3.**

Total number of properties for the input of the RNN. The input size matrix was 31X8, where each column was assigned to one property.

## 3. Results

Our RNN strategy was applied to the processed database that is outlined in Section 2.1. The goal was to predict the presence or absence of lysine glycation in the peptides of the independent test set. The results of this analysis consist of the quantitative values corresponding to the five metrics described in Section 2.1.

Consider Case 1 for the property IEP: the test set is composed of a list of 1000 peptides, where for each one of these, the RNN will assign a probability that there is glycation and another one that there is no glycation, totaling between them a 100% probability. Thus, a list of labels corresponding to the answer of whether there is glycation (represented by a 1) or no glycation (represented by a 0) has been built, where on the respective resulting quantitative values for the entire test set, each of the five metrics defined in Section 2.1 was calculated. If the same process is run a total of 20 times and the values obtained for each metric are averaged, a more reliable value of the RNN performance can be obtained, which will be linked to the probability of glycation or nonglycation on the respective peptide. This same procedure was applied for the rest of the properties, as well as for all the subcases of Case 2 and Case 3. In this way, a global evaluation and comparison of the multiple subcases studied in this work can be obtained. The label with the highest probability of glycation will be considered further.

**Figure 3:**
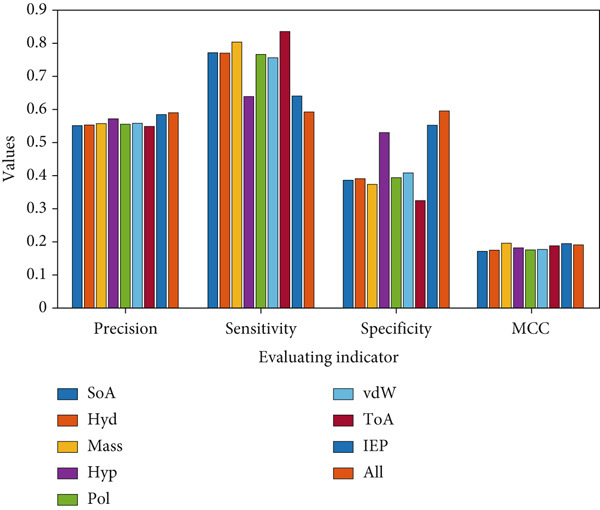
Performance metrics for Case 1 and Case 3. The four histograms correspond to the application of the metrics (Evaluating indicator): precision, sensitivity, specificity, and MCC. Each histogram shows the results for Case 1 (where all eight properties were tested individually) in its first eight columns and for Case 3 in the last column (where all eight properties were tested together). Mean values of each metric are shown.

**Figure 4:**
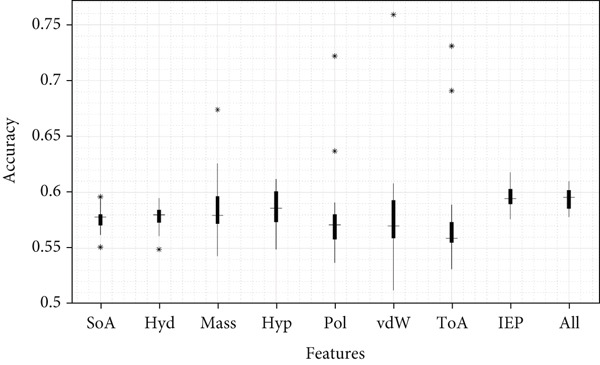
Detailed analysis of the accuracy metric for Case 1 and Case 3. The *x*‐axis lists the eight properties analyzed in Case 1 plus the combination of the eight properties (features) for a single analysis corresponding to Case 3 (all properties combined). The *y*‐axis shows the values achieved for the accuracy metric. Median values are indicated by horizontal lines; the ∗ symbol denotes outliers.

**Figure 5:**
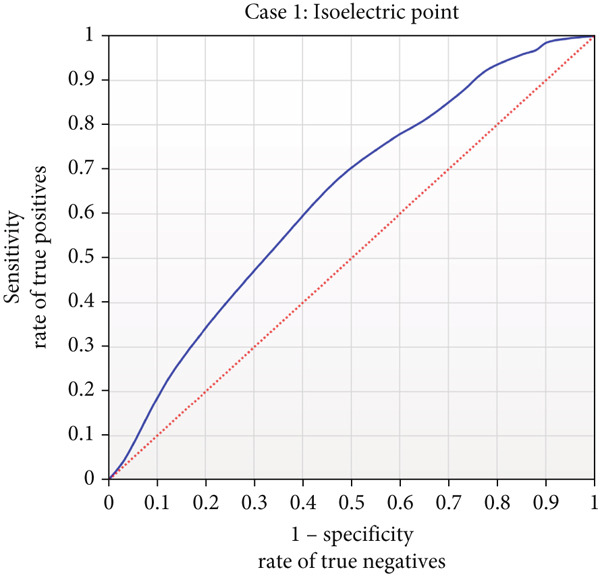
Receiver operating characteristic curve for IEP. ROC curve for the IEP as a physicochemical property to be used in the determination of the presence of glycation (Case 1). The blue curve shows the RNN results for the test set over Case 1 using the IEP as a physicochemical input. The dotted red line indicates the behavior of a purely random result.

Figures 3 and 4, Figures S1–S4, and Table S3 show the results obtained for Case 1 with respect to each of the metrics studied. It can be observed that the IEP is the physicochemical property that yields the highest values for the metrics Acc (0.596), Pre (0.584), and Spe (0.553). For the case of sensitivity, the highest value was 0.835, corresponding to the ToA; similarly, for the MCC, the highest value reached was 0.196, corresponding to the mass. Regarding the lowest values, for accuracy and MCC, the respective values of 0.576 (ToA/SoA) and 0.172 (Hyp/SoA) were obtained. A detailed analysis of the precision, sensitivity, specificity metrics, and the MCC for Case 1 is shown in Figures S1–S4. The ROC curve for IEP is presented in Figure 5; the blue line represents the result for the RNN, and the dotted line is a random result.

**Figure 6:**
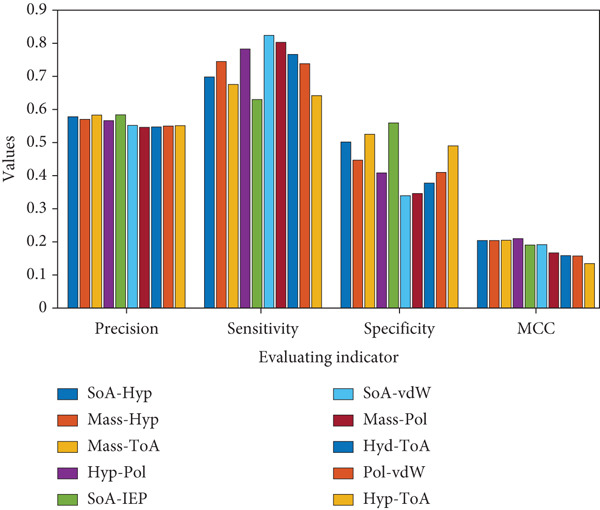
Performance metrics for selected Case 2 candidates. The four histograms correspond to the application of the metrics (evaluating indicator): precision, sensitivity, specificity, and MCC. Each histogram shows the results for the strongest (SoA + Hyp, Mass + Hyp, Mass + ToA, Hyp + Pol, SoA + IEP) and weakest (SoA + vdW, Mass + Pol, Hyd + ToA, Pol + vdW, Hyp + ToA) candidates of the accuracy metric. Mean values of each metric are shown.

**Figure 7:**
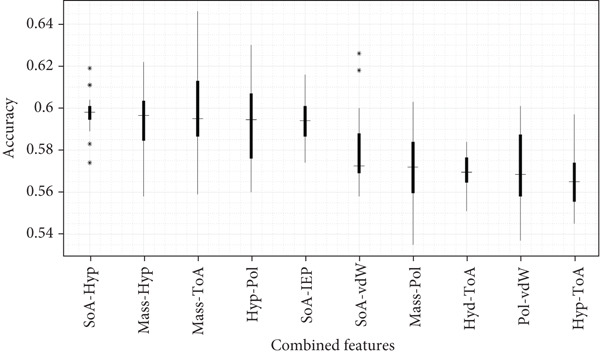
Detailed analysis of the accuracy metric for selected Case 2 candidates. Box plots display the accuracy distributions for the strongest (SoA + Hyp, Mass + Hyp, Mass + ToA, Hyp + Pol, SoA + IEP) and weakest (SoA + vdW, Mass + Pol, Hyd + ToA, Pol + vdW, Hyp + ToA) properties (features). Median values are indicated; the ∗ symbol denotes outliers.

**Figure 8:**
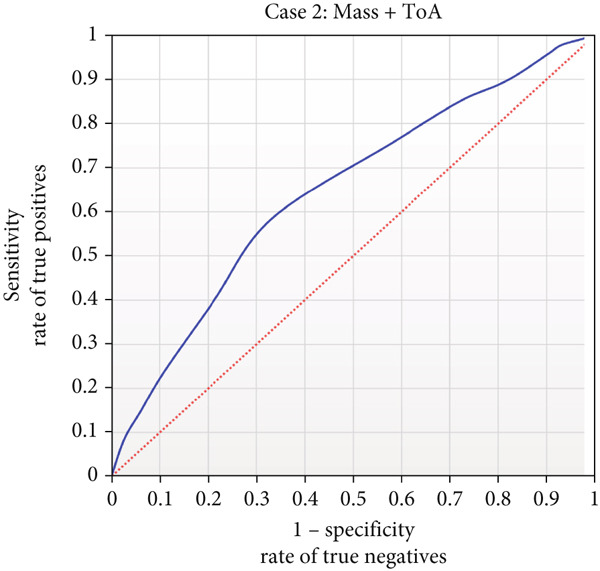
Receiver operating characteristic curve for mass and ToA. ROC curve for the combination of mass and ToA as physicochemical properties to be used in the determination of the presence of glycation (Case 2). The blue curve shows the RNN results for the test set over Case 1 when using the above combination as physicochemical input information. The dotted red line indicates the behavior that a purely random result would have.

For Case 2, the combination of both mass and ToA presented the highest accuracy value of 0.599 and in precision with a value of 0.583, respectively (Figures 6 and 7 and Table S4). It is noteworthy to point out that mass was the property that presented the highest values in the metrics applied after IEP for Case 1 and that it was found in two of the five best combinations of Case 2. Other properties that showed a notable effect on the performance of the RNN for this case were IEP, SoA, and Hyp. A detailed analysis of the accuracy for all property combinations of Case 2 is shown in Figure S5. Overall, for Case 2, only a slight improvement is achieved when compared to Case 1. Thus, we cannot state that combining three or more physicochemical properties will increase the performance of the RNN substantially, considering the contrast between the increase in computational complexity and the improvement in performance. The ROC curve for the case mass + ToA is presented in Figure 8; the blue line is the output of the RNN, and the dotted line is a random line.

**Figure 9:**
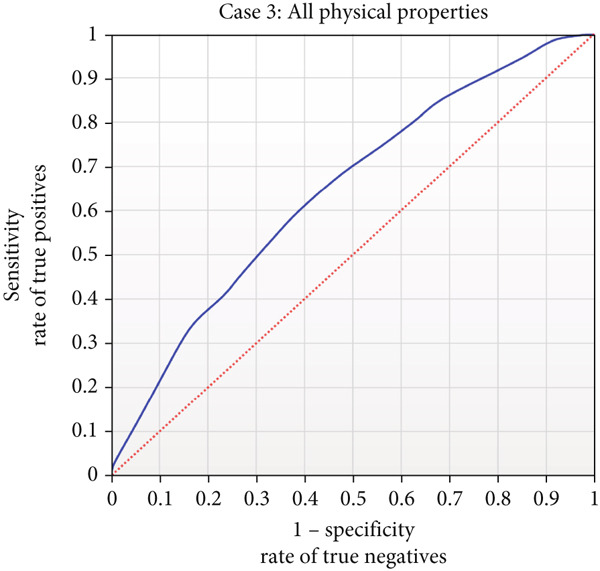
Receiver operating characteristic curve for Case 3. ROC curve for the combination of the eight physicochemical properties to be used in the determination of the presence of glycation (Case 3). The blue curve shows the RNN results for the test set over Case 3 when all physicochemical properties in combination are used as physicochemical input information. The dotted red line indicates the behavior that a purely random result would have.

For Case 3 (Figures 3 and 4 and Table S5), it is observed that the values obtained for all metrics were similar to those reported for both Case 2 and Case 1. As already mentioned, this infers that adding more physicochemical properties does not necessarily improve accuracy but can result in the opposite, generating more complexity to the learning of the RNN, so this requires greater complexity in the RNN and, therefore, greater computational power. This is the reason why, in the present work, combinations of three or more physicochemical properties are not included, considering that such cases imply the execution of many studies subcases (corresponding to each of the possible combinations), which, in preliminary tests, did not show an acceptable improvement in the performance of the RNN. The ROC curve for Case 3 is presented in Figure 9.

## 4. Discussion

In this work, we used an RNN in conjunction with eight physicochemical properties of amino acids to predict the presence of glycated lysine residues in protein sequences. Our results show that of all these properties, the one that shows the highest accuracy of prediction when only one property is analyzed is given by the IEP, followed by mass and Hyp.

When two properties are considered simultaneously, it was found that the combination of mass and ToA is the most important in determining the presence of lysine glycation. As was outlined in previous research [39], this finding demonstrates that the occurrence of glycation results from the combination of several physicochemical factors. This observation was already raised by Sjoblom et al. [24] in suggesting some properties that could have considerable weight for arginine glycation, such as the clustering of acidic residues decreasing glycation levels while the presence of a single negative charge may be important for glycation success. However, it is important to note that, from the present investigation, and as noted in the three case studies, this does not imply that combining all the physicochemical information available will result in an improved estimation of glycation likelihood.

Several approaches focused on identifying the presence or absence of glycation of proteinaceous lysine residues utilizing methodologies based on machine learning. The present work differs from previous studies in an important way, that is, how the database is processed to apply machine learning techniques. In most of the former works, the database of thousands of amino acid sequences, once filtered, had been subjected to algorithms for the extraction of properties that allow the determination of the presence or absence of glycation, such as amino acid composition, encoding based on grouped weight, or the *K*‐nearest neighbor feature (e.g., Glypre [31], PredGly [29], and BERT‐Kgly [34]). Our work does not employ any of these algorithms but instead focuses on replacing amino acid letters with numerical values of their different physicochemical properties. Because of this, it is not possible to make a direct comparison of the results of this project with other studies regarding the presence of glycation in lysine for an independent test. A correct comparison (such as the one performed by Liu et al. [34]) implies that each of the models to be compared must be applied to the same database. Because of this difficulty, it should be added that not all the models developed for the present line of research are available or work well, as specified in several studies [29, 34, 49]. To summarize, an important consideration regarding the methodologies presented in the field is that artificial intelligence and, specifically, models based on ANNs have gained significant strength and prominence in the last decade. In this regard, from the most current studies on the lysine glycation site, including the present work, RNNs based on LSTM algorithms represent one of the most promising methodologies.

Comparing the present results for lysine glycation with previous research [39] for arginine glycation, it is relevant to establish the cause of an apparent discrepancy in the results of both works. For lysine glycation, the most determining physicochemical property was IEP, followed by mass (Figures 3 and 4), whereas in Que‐Salinas et al. [39] IEP was among the weakest performers. In contrast, the mass had an average performance. The following considerations explain this contrasting result: firstly, although the focus in both works is similar, which is to discern which physicochemical properties have greater weight in the glycation process, the current work is focused on lysine, while in Que‐Salinas et al. [39], the study is focused on arginine. It is reasonable that different physicochemical properties play a bigger role in the glycation process of different amino acids. While the previous work focuses on estimating the value of the probability of glycation from the database published by Sjoblom et al. in 2018 [24], the present project focuses on classifying the presence or absence of glycation from the CPLM 4.0 database, so the approach of both methodologies is substantially different. It should be noted that the Case 2 combination SoA–ToA shows balanced sensitivity and specificity, indicating it performs consistently across classes.

Regarding the ratio of false positives and true negatives in our results, it is relevant to observe the “specificity” metric, which expresses the proportion of nonglycation sites estimated by the RNN that are real nonglycation sites. As shown in Figures 3 and 6, the specificity generally presented values below 0.5 and 0.4 for some of the results of Case 1. In the RNN calibration process, we have prioritized (through a 10‐fold cross‐validation) those models that allowed us to obtain a higher value in the sensitivity, which means we consider that the most important result is how capable the RNN selects the glycation sites. Consequently, the sensitivity reaches high values in general (higher than 0.8 in some subcases). This implies that the RNN is working well to clearly discern lysine glycation sites.

For Case 3, when all physicochemical properties are used together, despite a slightly lower accuracy, the results obtained for all metrics suggest that this case could be more robust toward a future application, especially if there is an imbalance in the data sets. More studies are needed to contrast these results in determining the effect of the physicochemical properties of amino acids near the glycation site on the glycation process. The properties of neighboring amino acids that play a significant role in the glycation process of lysine residues in proteins are both the IEP and mass.

While our study presents a robust framework for the prediction of lysine glycation, it comes with some limitations that need to be discussed. Firstly, the dataset used in CPLAM 4.0 is influenced by experimental biases inherent to MS‐based proteomics, such as incomplete peptide detection and underrepresentation of membrane or hydrophobic proteins. Proteomic coverage is incomplete, with many lysine residues unassessed due to technical constraints or low protein abundance. Biological source bias is also evident, with a dominant focus on human and mouse samples. Moreover, while the database includes annotations for numerous lysine modifications, many sites lack direct functional validation or disease relevance, limiting biological interpretation. Literature bias further skews the dataset toward well‐studied proteins and PTMs that are more likely to be published. As a result, CPLM 4.0, while extensive, reflects the current state of experimental PTM research rather than a comprehensive or unbiased view of lysine modification biology.

Secondly, glycation is not a strictly binary process, despite the common classification of potential sites (i.e., lysine, arginine, and cysteine) as “glycated” or “nonglycated.” These sites can undergo various covalent modifications, not just glycation. Factors such as the type and concentration of electrophiles (e.g., MGO), their half‐life in the cell, and the local structural context of the protein will affect side chain reactivity. These considerations are not reflected in our current approach, which relies solely on peptide sequence data.

## 5. Conclusions

This work outlines a comprehensive conceptual framework for predicting the susceptibility of proteinaceous lysine residues to glycation. A dataset of protein lysine modifications, filtered to exclude redundancy using the CD‐HIT method, has been utilized. The database used is relatively large but does not contain quantitative data. It is also heterogeneous regarding methods that various laboratories have utilized to measure glycation in diverse biological systems. This contrasts with the relatively small quantitative data set on arginine glycation of short synthetic peptides reported by Sjoblom et al. [24], which formed the basis of our previous article [39]. Employing an RNN for lysine glycation classification allowed the identification of properties that are suggested to play an important role in lysine glycation. Specifically, by utilizing the IEP as the sole physicochemical property for peptide characterization, a 59.6% accuracy in predicting lysine glycation was achieved. Furthermore, integrating two properties, mass and ToA, increased accuracy to 59.9%. Employing all eight properties led to a slightly reduced accuracy of 59.4%. The results obtained reflect the relevant relationships between the physicochemical properties and the glycation process, meaning that the sequential structure of the peptide plays an important role. Our approach is designed to contribute to the existing landscape of the lysine‐residue glycation estimation algorithms and to expand and enhance this landscape substantively. Although a perfect tool for unfailing predictions does not exist yet, our approach using eight physicochemical properties of amino acids neighboring the glycation site to determine its modification probability is pointing to a promising direction. The main challenge in improving the algorithm is the standardization of experimental determination of glycation sites in proteins, because different laboratories employ different methodologies and setups. This also includes the reporting of the glycation occurrence, whether it is quantitative (e.g., expressed as a percentage value) or qualitative.

## Ethics Statement

The authors have nothing to report.

## Disclosure

A preprint version of this article has previously been published [50]. All authors have read and agreed to the submitted version of the manuscript.

## Conflicts of Interest

The authors declare no conflicts of interest.

## Author Contributions

Conceptualization: U.Q.‐S., D.M.‐P., and C.Q.S. Formal analysis: U.Q.‐S. and D.M.‐P. Investigation: U.Q.‐S., D.M.‐P., G.M.M., A.D.R.‐F., P.A.‐L., and C.Q.S. Methodology: U.Q.‐S. Project administration: A.D.R.‐F. and C.Q.S. Supervision: C.Q.S. Validation: U.Q.‐S. and D.M.‐P. Visualization: D.M.‐P. Writing—original draft: U.Q.‐S., D.M.‐P., and C.Q.S. Writing—review and editing: U.Q.‐S., D.M.‐P., A.D.R.‐F., G.M.M., and C.Q.S.

## Funding

This study was supported by the Consejo Nacional de Humanidades, Ciencia y Tecnología (CONAHCyT) (I1200/224/2021).

## Supporting information


**Supporting Information** Additional supporting information can be found online in the Supporting Information section. Table S1: Numerical values corresponding to the eight physicochemical properties of amino acids used in this study. Table S2: Data set with the number of glycation sites. Table S3: The mean values of the classification results for one property (Case 1). Table S4: Performance of classification for the combination of two properties (Case 2). Table S5: Performance of classification for the combination of all eight properties (Case 3). Figure S1: Detailed analysis of the precision for Case 1 and Case 3 (All). Figure S2: Detailed analysis of the specificity for Case 1 and Case 3 (All). Figure S3: Detailed analysis of the sensitivity for Case 1 and Case 3 (All). Figure S4: Detailed analysis of the MCC for Case 1 and Case 3 (All). Figure S5: Detailed analysis of the accuracy for Case 2.

## Data Availability

All data is available in the supporting information.
