# Supplementary material for: Addressing the Problem of Lysine Glycation Prediction in Proteins via Recurrent Neural Networks
Source: Biomed Res Int. 2025 Sep 15;2025:2426944. doi: 10.1155/bmri/2426944 (PMC12436683; doi:10.1155/bmri/2426944)
Supplement: Supplementary file 1 — Supporting Information Additional supporting information can be found online in the Supporting Information section. Table S1: Numerical values corresponding to the eight physicochemical properties of amino acids used in this study. Table S2: Data set with the number of glycation sites. Table S3: The mean values of the classification results for one property (Case 1). Table S4: Performance of classification for the combination of two properties (Case 2). Table S5: Performance of classification for the combination of all eight properties (Case 3). Figure S1: Detailed analysis of the precision for Case 1 and Case 3 (All). Figure S2: Detailed analysis of the specificity for Case 1 and Case 3 (All). Figure S3: Detailed analysis of the sensitivity for Case 1 and Case 3 (All). Figure S4: Detailed analysis of the MCC for Case 1 and Case 3 (All). Figure S5: Detailed analysis of the accuracy for Case 2. [file BMRI-2025-2426944-s001.docx]

Addressing the problem of lysine glycation prediction in proteins via Recurrent Neural Networks

-Supplementary material-

Ulices Que-Salinas^1^, Dulce Martinez-Peon^2^, Gerardo Maximiliano Mendez^2^,

P. Argüelles-Lucho^3^, Angel D. Reyes-Figueroa^4,5^,

Christian Quintus Scheckhuber^6,*^

1 Centro de Ciencias de la Tierra, Universidad Veracruzana, Xalapa 91090, VER, México

2 Tecnológico Nacional de México/ Instituto Tecnológico de Nuevo León, Departamento de Ingeniería Eléctrica y Electrónica, Av. Eloy Cavazos 2001, Guadalupe 67170, NL, México

3 Tecnológico Nacional de México, Instituto Tecnológico de Veracruz, Calzada Miguel Ángel de Quevedo 2779, Veracruz 91860, VER, México

4 Consejo Nacional de Ciencia y Tecnología, Av. Insurgentes Sur 1582, Col. Crédito Constructor, Benito Juárez, Mexico City 03940, DF, México

5 Centro de Investigación en Matemáticas Unidad Monterrey, Parque de Investigación e Innovación Tecnológica (PIIT), Av. Alianza Centro No. 502, Apodaca 66628, NL, México

6 Tecnológico de Monterrey, Escuela de Ingeniería y Ciencias, Av. Eugenio Garza Sada 2501 Sur, Col. Tecnológico, Monterrey 64700, NL, México

* Correspondence – email: c.scheckhuber@tec.mx

**Supplementary methods**

**Table S1** **Numerical values corresponding to the eight physicochemical properties of amino acids used in this study** To form the vectors corresponding to each of the 6830 sequences of 31 amino acids, the letter which represents each amino acid is treated as a label that is exchanged for the numerical value based on the chosen physicochemical property. The first column contains the names of the amino acids, while the next two tables contain their abbreviations. From the fourth column onwards, the numerical values of the 20 amino acids are presented for each of the eight physicochemical properties.

| **Full Name** | **Abbreviation** | | **Properties** | | | | | | | |
| --- | --- | --- | --- | --- | --- | --- | --- | --- | --- | --- |
|  | 3 Letter | 1 Letter | Structure of the amino acid sequence (SoA) | Hydropathy (Hyd) | Mass (Mass) | Hydrophobicity (Hyp) | Polarizability (Pol) | Normalized van der Waals volume (vdW) | Torsion angle (ToA) | Isoelectric point (IEP) |
| Alanine | Ala | A | 1 | 1.8 | 89 | 0.31 | 0.05 | 1 | 4.76 | 6.11 |
| Arginine | Arg | R | -12 | -4.5 | 174 | -1.01 | 0.29 | 6.13 | 4.3 | 10.74 |
| Asparagine | Asn | N | -7 | -3.5 | 132 | -0.6 | 0.13 | 2.95 | 3.64 | 6.52 |
| Aspartate | Asp | D | -8 | -3.5 | 133 | -0.77 | 0.11 | 2.78 | 3.69 | 2.95 |
| Cysteine | Cys | C | 3 | 2.5 | 121 | 1.54 | 0.13 | 2.43 | 3.67 | 6.35 |
| Glutamine | Gln | Q | -9 | -3.5 | 146 | -0.22 | 0.18 | 3.95 | 4.54 | 5.65 |
| Glutamate | Glu | E | -10 | -3.5 | 147 | -0.64 | 0.15 | 3.78 | 5.48 | 3.09 |
| Glycine | Gly | G | 0 | -0.4 | 75 | 0 | 0 | 0 | 3.77 | 6.07 |
| Histidine | His | H | -6 | -3.2 | 155 | 0.13 | 0.23 | 4.66 | 2.84 | 7.69 |
| Isoleucine | Ile | I | 7 | 4.5 | 131 | 1.8 | 0.19 | 4 | 4.81 | 6.04 |
| Leucine | Leu | L | 5 | 3.8 | 131 | 1.7 | 0.19 | 4 | 4.79 | 6.04 |
| Lysine | Lys | K | -11 | -3.9 | 146 | -0.99 | 0.22 | 4.77 | 4.27 | 9.99 |
| Methionine | Met | M | 2 | 1.9 | 149 | 1.23 | 0.22 | 4.43 | 4.25 | 5.71 |
| Phenylalanine | Phe | F | 4 | 2.8 | 165 | 1.79 | 0.29 | 5.89 | 4.31 | 5.67 |
| Proline | Pro | P | -5 | -1.6 | 115 | 0.72 | 0.12 | 2.72 | 2,84 | 6.8 |
| Serine | Ser | S | -2 | -0.8 | 105 | -0.04 | 0.06 | 1.6 | 3.83 | 5.7 |
| Threonine | Thr | T | -1 | -0.7 | 119 | 0.26 | 0.11 | 2.6 | 3.87 | 5.6 |
| Tryptophan | Trp | W | -3 | -0.9 | 204 | 2.25 | 0.41 | 8.08 | 4.75 | 5.94 |
| Tyrosine | Tyr | Y | -4 | -1.3 | 181 | 0.96 | 0.3 | 6.47 | 4.3 | 5.66 |
| Valine | Val | V | 6 | 4.2 | 117 | 1.22 | 0.14 | 3 | 4.86 | 6.02 |

**Table S2 Data set with the number of glycation sites** Data is organized in ten folds with 6830 sequences each. Folds 1 to 4 have the same number of negative and positive labels. Folds 5 to 10 have 3416 positive labels and 3414 negative labels. According to Wang et al. 2021* the database is balanced because the number of both instances is almost similar; for an unbalanced database, one of the classes must be significantly different from the other.

|  | **Positive** | **Negative** |
| --- | --- | --- |
| Fold 1 | 3415 | 3415 |
| Fold 2 | 3415 | 3415 |
| Fold 3 | 3415 | 3415 |
| Fold 4 | 3415 | 3415 |
| Fold 5 | 3416 | 3414 |
| Fold 6 | 3416 | 3414 |
| Fold 7 | 3416 | 3414 |
| Fold 8 | 3416 | 3414 |
| Fold 9 | 3416 | 3414 |
| Fold 10 | 3416 | 3414 |

* L. Wang, M. Han, X. Li, N. Zhang and H. Cheng, "Review of Classification Methods on Unbalanced Data Sets," in IEEE Access, vol. 9, pp. 64606-64628, 2021, doi: 10.1109/ACCESS.2021.3074243.

**Supplementary results**

The results are presented below in tables containing the quantitative values, as well as graphs to allow making comparisons. For case 1, each of the eight physicochemical properties was selected separately to run the numerical algorithm, and the values corresponding to the five metrics were calculated from the results obtained by the RNN (Table S3). For case 2, two of the physicochemical properties were selected to run the numerical algorithm and the values corresponding to the five metrics were calculated from the results obtained by the RNN (Table S4). For case 3, all the physicochemical properties corresponding to case 1 were selected to run the numerical algorithm and the values corresponding to the five metrics were calculated from the results obtained by the RNN (Table S5).

**Table S3** **The mean values of the classification results for one property (case 1)** The data have been sorted in descending order of accuracy. The values in bold text are the highest for each metric. Acc: accuracy, Pre: precision, Sen: sensitivity, Spe: specificity, MCC: Matthews Correlation Coefficient.

| **Properties** | **Acc** | **Pre** | **Sen** | **Spe** | **MCC** |
| --- | --- | --- | --- | --- | --- |
| **IEP** | **0.5961** | **0.5839** | 0.6399 | **0.5534** | 0.1947 |
| **Mass** | 0.5862 | 0.5572 | 0.8036 | 0.374 | **0.1963** |
| **Hyp** | 0.5841 | 0.5714 | 0.6391 | 0.5305 | 0.1713 |
| **vdW** | 0.5807 | 0.5579 | 0.7568 | 0.4087 | 0.1775 |
| **Hyd** | 0.5785 | 0.553 | 0.7695 | 0.392 | 0.175 |
| **Pol** | 0.5782 | 0.5551 | 0.7661 | 0.3947 | 0.1752 |
| **SoA** | 0.5761 | 0.5507 | 0.7715 | 0.3855 | 0.1716 |
| **ToA** | 0.5761 | 0.5486 | **0.8345** | 0.3237 | 0.1879 |

**Table S4** **Performance of classification for the combination of two properties (case 2)** The data have been sorted in descending order of accuracy. The values in bold text are the highest for each metric. Acc: accuracy, Pre: precision, Sen: sensitivity, Spe: specificity, MCC: Matthews Correlation Coefficient.

| **Two properties** | **Acc** | **Pre** | **Sen** | **Spe** | **MCC** |
| --- | --- | --- | --- | --- | --- |
| Mass-ToA | **0.59935** | 0.582911 | 0.675202 | 0.525296 | 0.204667 |
| SoA-Hyp | 0.59785 | 0.577849 | 0.696761 | 0.501285 | 0.203254 |
| SoA-IEP | 0.59405 | 0.583299 | 0.630162 | 0.558794 | 0.190302 |
| Mass-Hyp | 0.59385 | 0.570103 | 0.74413 | 0.447134 | 0.203743 |
| Hyp-Pol | 0.59315 | 0.565774 | 0.782287 | 0.408498 | **0.209863** |
| Hyp-vdW | 0.58965 | 0.565676 | 0.771356 | 0.412253 | 0.202146 |
| ToA-IEP | 0.5896 | 0.574368 | 0.660425 | 0.520455 | 0.184202 |
| Hyp-IEP | 0.58835 | 0.574858 | 0.64251 | 0.535474 | 0.179306 |
| Mass-IEP | 0.58785 | **0.58331** | 0.582389 | 0.593182 | 0.175917 |
| Hyd-IEP | 0.58685 | 0.574435 | 0.633907 | 0.540909 | 0.176063 |
| Mass-vdW | 0.586 | 0.557809 | 0.787247 | 0.389526 | 0.192511 |
| Hyd-Hyp | 0.585 | 0.568719 | 0.664474 | 0.507411 | 0.174965 |
| vdW-ToA | 0.584 | 0.569207 | 0.676215 | 0.493972 | 0.175731 |
| Hyd-Pol | 0.58295 | 0.55725 | 0.771964 | 0.398419 | 0.185834 |
| Hyd-Mass | 0.5826 | 0.555974 | 0.784312 | 0.385672 | 0.187493 |
| Pol-IEP | 0.581 | 0.570902 | 0.616802 | 0.546047 | 0.164265 |
| SoA-Hyd | 0.57985 | 0.551462 | 0.802834 | 0.362154 | 0.184381 |
| SoA-vdW | 0.57965 | 0.551396 | 0.825405 | 0.339723 | 0.192076 |
| SoA-Pol | 0.57945 | 0.550346 | **0.826316** | 0.338439 | 0.191261 |
| SoA-Mass | 0.5792 | 0.552159 | 0.794028 | 0.369466 | 0.182328 |
| Hyd-vdW | 0.5785 | 0.550321 | 0.808198 | 0.354249 | 0.183056 |
| vdW-IEP | 0.57715 | 0.566692 | 0.615486 | 0.539723 | 0.156209 |
| Pol-ToA | 0.57685 | 0.562086 | 0.668117 | 0.487747 | 0.15987 |
| SoA-ToA | 0.5754 | 0.552194 | 0.75081 | **0.75081** | 0.168006 |
| Pol-vdW | 0.5722 | 0.550802 | 0.73836 | 0.40998 | 0.157936 |
| Mass-Pol | 0.57165 | 0.545927 | 0.802429 | 0.346344 | 0.167115 |
| Hyd-ToA | 0.5699 | 0.546433 | 0.766397 | 0.378063 | 0.159223 |
| Hyp-ToA | 0.56505 | 0.552098 | 0.641397 | 0.490514 | 0.134274 |

**Table S5** **Performance of classification for the combination of all eight properties (case 3)** Acc: accuracy, Pre: precision, Sen: sensitivity, Spe: specificity, MCC: Matthews Correlation Coefficient.

| **Properties** | **Acc** | **Pre** | **Sen** | **Spe** | **MCC** |
| --- | --- | --- | --- | --- | --- |
| **All** | 0.5944 | 0.59 | 0.5925 | 0.5961 | 0.1903 |

For a detailed analysis of the results, the following figures S1 to S5 contain the values obtained for each of the calculated metrics. For simplicity, the values of the metrics for cases 1 and 3 are shown in the same figure.


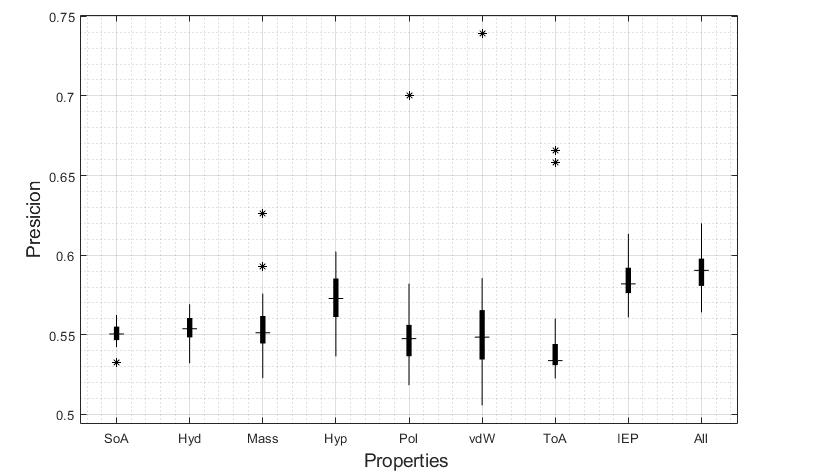


**Figure S1 Detailed analysis of the precision for case 1 and case 3 (All)** The x axis lists the eight analyzed properties. Median values are indicated by horizontal lines, the * symbol denotes outliers.


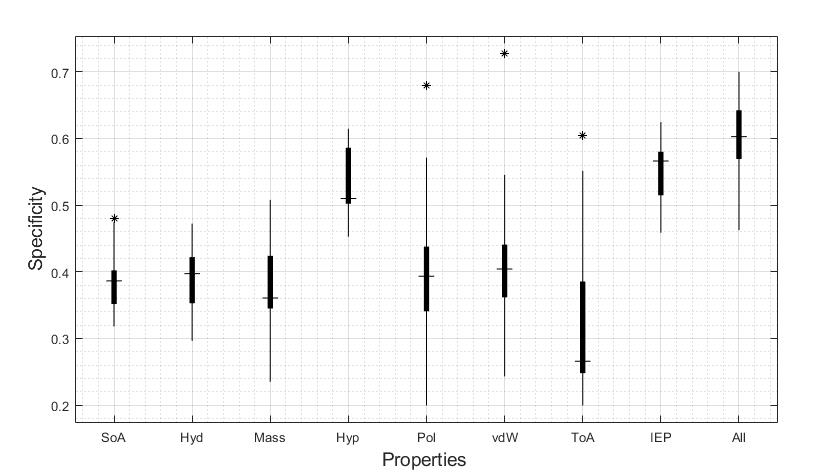


**Figure S2 Detailed analysis of the specificity for case 1 and case 3 (All)** The x axis lists the eight analyzed properties. Median values are indicated by horizontal lines, the * symbol denotes outliers.


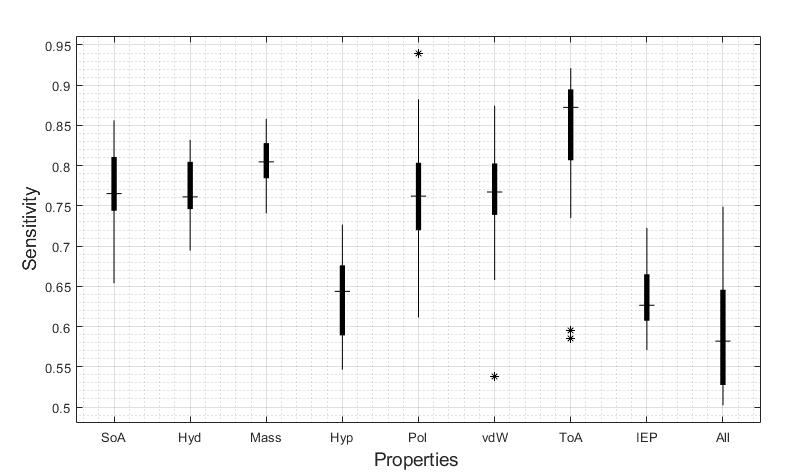


**Figure S3 Detailed analysis of the sensitivity for case 1 and case 3 (All)** The x axis lists the eight analyzed properties. Median values are indicated by horizontal lines, the * symbol denotes outliers.


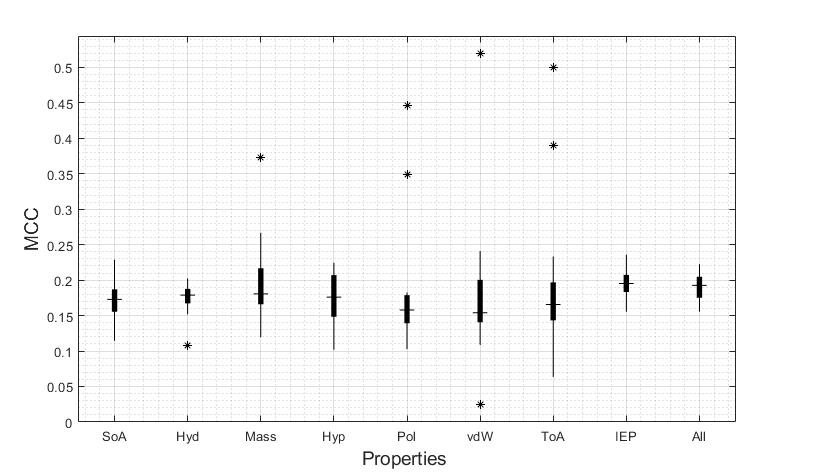


**Figure S4 Detailed analysis of the MCC for case 1 and case 3 (All)** The x axis lists the eight analyzed properties. Median values are indicated by horizontal lines, the * symbol denotes outliers.


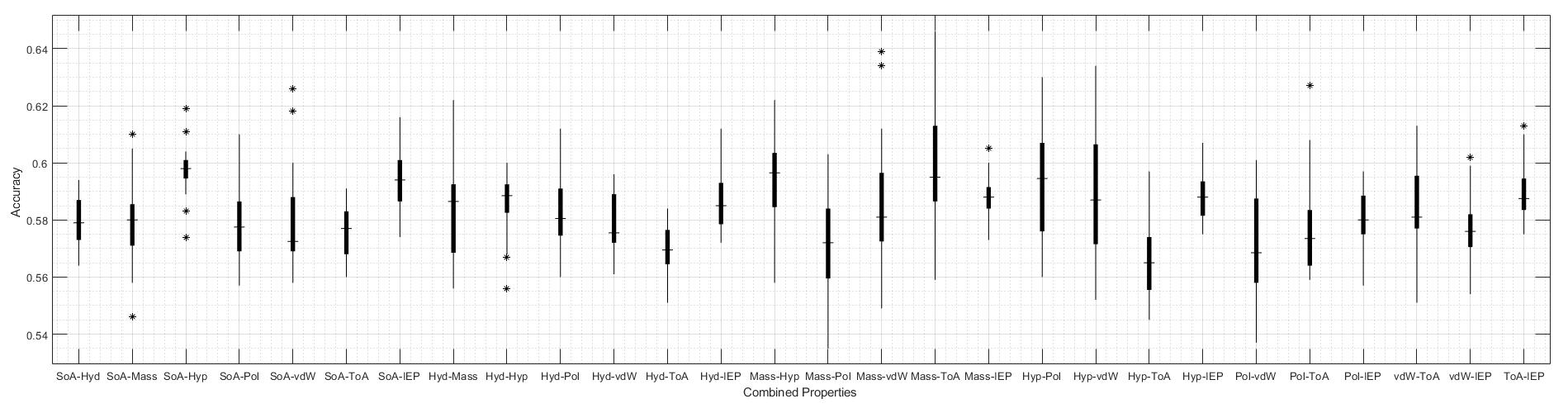


**Figure S5 Detailed analysis of the accuracy for case 2** The x axis lists the combined properties. Median values are indicated by horizontal lines, the * symbol denotes outliers.
